# Glycolytic fast-twitch muscle fiber restoration counters adverse age-related changes in body composition and metabolism

**DOI:** 10.1111/acel.12153

**Published:** 2013-09-17

**Authors:** Yuichi Akasaki, Noriyuki Ouchi, Yasuhiro Izumiya, Barbara L Bernardo, Nathan K LeBrasseur, Kenneth Walsh

**Affiliations:** 1 Molecular Cardiology Whitaker Cardiovascular Institute, Boston University School of MedicineBoston, MA, 02118, USA; 2 Department of Cardiovascular, Respiratory and Metabolic Medicine, Graduate School of Medical and Dental Sciences, Kagoshima University8-35-1 Sakuragaoka, Kagoshima, 890-8520, Japan; 3 Department of Molecular Cardiology, Nagoya University School of Medicine65 Tsurumai-cho Showa-ku, Nagoya, 466-8550, Japan; 4 Department of Cardiovascular Medicine, Graduate School of Medical Sciences, Kumamoto UniversityKumamoto, 860-8556, Japan; 5 Pfizer Global Research and DevelopmentGroton, CT, 06340, USA; 6 Robert and Arlene Kogod Center on Aging, Mayo ClinicRochester, MN, 55905, USA

**Keywords:** adipose tissue, diabetes, exercise, mTOR, sarcopenia, type IIb muscle

## Abstract

Aging is associated with the development of insulin resistance, increased adiposity, and accumulation of ectopic lipid deposits in tissues and organs. Starting in mid-life there is a progressive decline in lean muscle mass associated with the preferential loss of glycolytic, fast-twitch myofibers. However, it is not known to what extent muscle loss and metabolic dysfunction are causally related or whether they are independent epiphenomena of the aging process. Here, we utilized a skeletal-muscle-specific, conditional transgenic mouse expressing a constitutively active form of Akt1 to examine the consequences of glycolytic, fast-twitch muscle growth in young vs. middle-aged animals fed standard low-fat chow diets. Activation of the Akt1 transgene led to selective skeletal muscle hypertrophy, reversing the loss of lean muscle mass observed upon aging. The Akt1-mediated increase in muscle mass led to reductions in fat mass and hepatic steatosis in older animals, and corrected age-associated impairments in glucose metabolism. These results indicate that the loss of lean muscle mass is a significant contributor to the development of age-related metabolic dysfunction and that interventions that preserve or restore fast/glycolytic muscle may delay the onset of metabolic disease.

## Introduction

Aging is a primary risk factor for insulin resistance and the pathogenesis of type 2 diabetes mellitus (T2DM). In the United States, the prevalence of T2DM sharply increases from ~4% of the adult population younger than 44 years of age to 27% of those older than 65 (Centers for Disease Control and Prevention, 2011). Statistically, the greatest incidence of newly diagnosed cases of diabetes occurs in middle-aged individuals (ages 45–64). This trend coincides with age-related changes in body composition that commence in mid-life, including loss of lean mass, increased visceral adiposity, and ectopic lipid accumulation in liver and skeletal muscle, recognized as powerful mediators of insulin resistance (Kohrt *et al*., 1993; Basu *et al*., 2003). The aging population and anticipated increase in the incidence of T2DM underscore the critical need for better understanding how diabetes is influenced by age-associated changes in body composition.

Loss of skeletal muscle mass is a hallmark of aging that starts in the fourth decade of life (Janssen *et al*., 2000) and reflects a preferential loss and atrophy of glycolytic, fast-twitch fibers (type IIx fibers in humans and type IIb fibers in rodents) (Larsson, 1983). Age-related loss of skeletal muscle mass and strength (sarcopenia) underlies age-associated decrements in physical performance, and presumably also has metabolic implications (Janssen & Ross, 2005). Skeletal muscle is the primary site of insulin-mediated glucose disposal, is the largest reservoir of glycogen in humans, and accounts for approximately 30% of resting energy expenditure. Therefore, it is plausible that changes in skeletal muscle mass contribute to the onset of insulin resistance and T2DM in middle-aged individuals. However, whether targeted restoration or maintenance of skeletal muscle mass can directly affect metabolic homeostasis and prevent T2DM has not been directly examined in an aging organism.

The PI3-kinase-/Akt-/mTOR-signaling pathway controls tissue growth, largely through its ability to control the size of individual cells (Shiojima & Walsh, 2006). In skeletal muscle, this signaling pathway is activated by anabolic stimuli including growth factors (i.e., IGF-1), resistance exercise, and nutritional inputs (LeBrasseur *et al*., 2011). Selective activation of this signaling pathway in muscle leads to an increase in the cross-sectional area of muscle fibers referred to as hypertrophy (Rommel *et al*., 2001; Takahashi *et al*., 2002). Conversely, inactivation of this pathway leads to the activation of a FOXO transcriptional program and the atrophy of muscle (Sandri *et al*., 2004; LeBrasseur *et al*., 2011). A number of studies have documented that aging leads to anabolic resistance: namely, a reduction in the responsiveness of the PI3K/Akt/mTOR pathway and downstream effectors to pro-growth interventions including growth factors and resistance exercise and, in turn, an attenuation of fiber hypertrophy (Fiatarone *et al*., 1990, 1994; McCartney *et al*., 1995; Welle *et al*., 1996; Hakkinen *et al*., 1998; Tamaki *et al*., 2000; Morris *et al*., 2004; Parkington *et al*., 2004; Funai *et al*., 2006; Haddad & Adams, 2006). However, causal evidence linking this signaling decline to age-dependent reductions in muscle mass is lacking. More importantly, it is unknown to what extent the loss of skeletal muscle mass directly contributes to systemic metabolic dysfunction that occurs upon aging. Put another way, it is not known whether muscle loss and insulin resistance are independent epiphenomena of the aging process, or if the loss of muscle mass is causally linked to the high incidence of insulin resistance and T2DM in middle-aged persons.

Using mice engineered to overexpress or ablate PGC-1α in muscle, a transcription factor that promotes mitochondrial biogenesis, a series of studies have reported mixed results regarding the involvement of oxidative muscle fibers in either metabolically challenged or aging mice (Benton *et al*., 2008; Choi *et al*., 2008; Wenz *et al*., 2009; Zechner *et al*., 2010; Finley *et al*., 2012). There is a paucity of information on the role of glycolytic muscle fibers in the control of these phenotypes. To address these issues, we developed a skeletal-muscle-specific, conditional transgenic mouse that expresses a constitutively activated form of Akt1 in skeletal muscle (Izumiya *et al*., 2008). In young mice, transgene activation leads to the hypertrophy of type IIb muscle fibers that are associated with an increase in strength, but not an increase in running performance. Thus, we reasoned this model is well suited to assess the role of age-dependent muscle loss on systemic metabolism because transgene activation leads to the selective growth of glycolytic fast-twitch fibers, that is, the fibers that are preferentially lost during the aging process (Lexell, 1995). Transgene activation in this model leads to an overall 5% increase in lean muscle mass (Izumiya *et al*., 2008), matching the modest loss of muscle caused by the early stages of aging. Furthermore, the transgene encodes an intracellular signaling protein in muscle (i.e., Akt1), rather than a circulating factor, permitting a rigorous assessment of the impact of muscle mass *per se* on systemic metabolic function.

Using this model, we analyzed whether activation of Akt1 in muscle fibers counters early age-associated changes in body composition and metabolism. At 1 year of age, mice on a standard chow diet lose approximately 5% lean muscle mass, and this is associated with an increase in adipose tissue mass, insulin resistance, and the development of hepatic steatosis. Akt1 transgene activation in aging mice led to the selective hypertrophy of type IIb fibers, restored lean muscle mass, improved metabolic parameters, and diminished the extent of hepatic steatosis. These data indicate that interventions to prevent or restore the age-related loss of lean muscle that occurs during mid-life can have a significant impact on systemic metabolic function.

## Results

### Blunted Akt activation by growth factor stimulation in muscle of middle-aged mice

**Figure 1:**
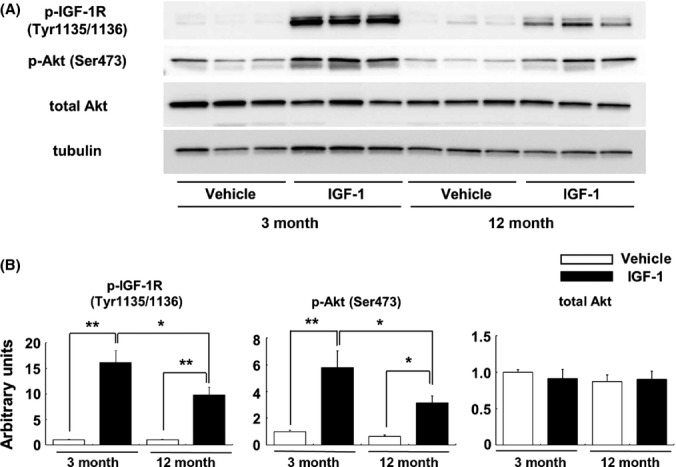
Response to anabolic stimulation is blunted by aging. (A) Western blot analysis was performed to assess phosphorylation of insulin growth factor-1 (IGF-1) receptor beta at tyrosine 1135/1136, Akt at serine 473 and total Akt in gastrocnemius muscle of 3- or 12-month-old mice (*n* = 6/group). Vehicle group was injected with sterile water, and the IGF-1 group was treated with human insulin growth factor-1. (B) Phosphorylation or protein levels were determined relative to tubulin by Image J software (*n* = 6/group). Results are presented as mean ± SEM. *P < 0.05, **P < 0.01.

To compare the responsiveness of younger and older skeletal muscles to an anabolic stimulus, either saline or 100 μg of recombinant insulin-like growth factor-1 (IGF-1) was administered by intramuscular injection into the gastrocnemius muscle of 3- or 12-month-old mice (*n* = 12/age group, *n* = 6/treatment). Muscles were harvested 60 min later and processed for analysis of protein abundance and phosphorylation. In younger mice, IGF-1 robustly increased the activating phosphorylation of the IGF-1 receptor (IGF-1R) and Akt (Fig. 1A). Delivery of IGF-1 to the muscle of middle-aged mice also increased phosphorylation of IGF-1R and Akt; however, the magnitudes of these changes at both the receptor and its downstream effector were significantly less than observed in younger mice (Fig. 1B). Total levels of Akt protein did not differ between experimental groups (Fig. 1B).

### Increasing fast/glycolytic fiber mass reverses age-associated changes in body composition

Because aging is associated with impaired Akt activation in muscle, we sought to compare the effects of acutely activating this signaling step in the skeletal muscle of younger and older mice using a doxycycline (DOX)-regulated double-transgenic system. This system was developed by crossing the ‘effector’ line, TRE-myrAkt1 (Shiojima *et al*., 2005), with the skeletal-muscle-specific ‘driver’ line, 1256[3Emut] MCK-rtTA that shows little or no activity in slow/oxidative (type I) muscle fibers or in cardiac muscle (Izumiya *et al*., 2008). Offspring containing both transgenes, referred to as double-transgenic (DTG) mice, exhibit robust Akt1 induction and the selective growth of type IIb-positive, fast/glycolytic myofibers in a subset of skeletal muscle groups when administered DOX in their drinking water (Izumiya *et al*., 2008). Transgene activation in this system had no observable effects on the distribution or size of type I or type IIa myofibers. To examine the effects of muscle-specific Akt1 transgene activation in young (3 month old) and older (12 month old) DTG mice, we administered DOX in their drinking water for 4 weeks. Control mice were MCK-rtTA single transgenic littermates that also received DOX. Consistent with previous data, there were no DOX-associated changes in skeletal muscle signaling or mass in control mice (Izumiya *et al*., 2008). DTG and control mice were treated in an identical manner and were fed a standard low-fat chow diet (18.6% protein, 44.2% carbohydrate, and 6.2% fat).

**Figure 2:**
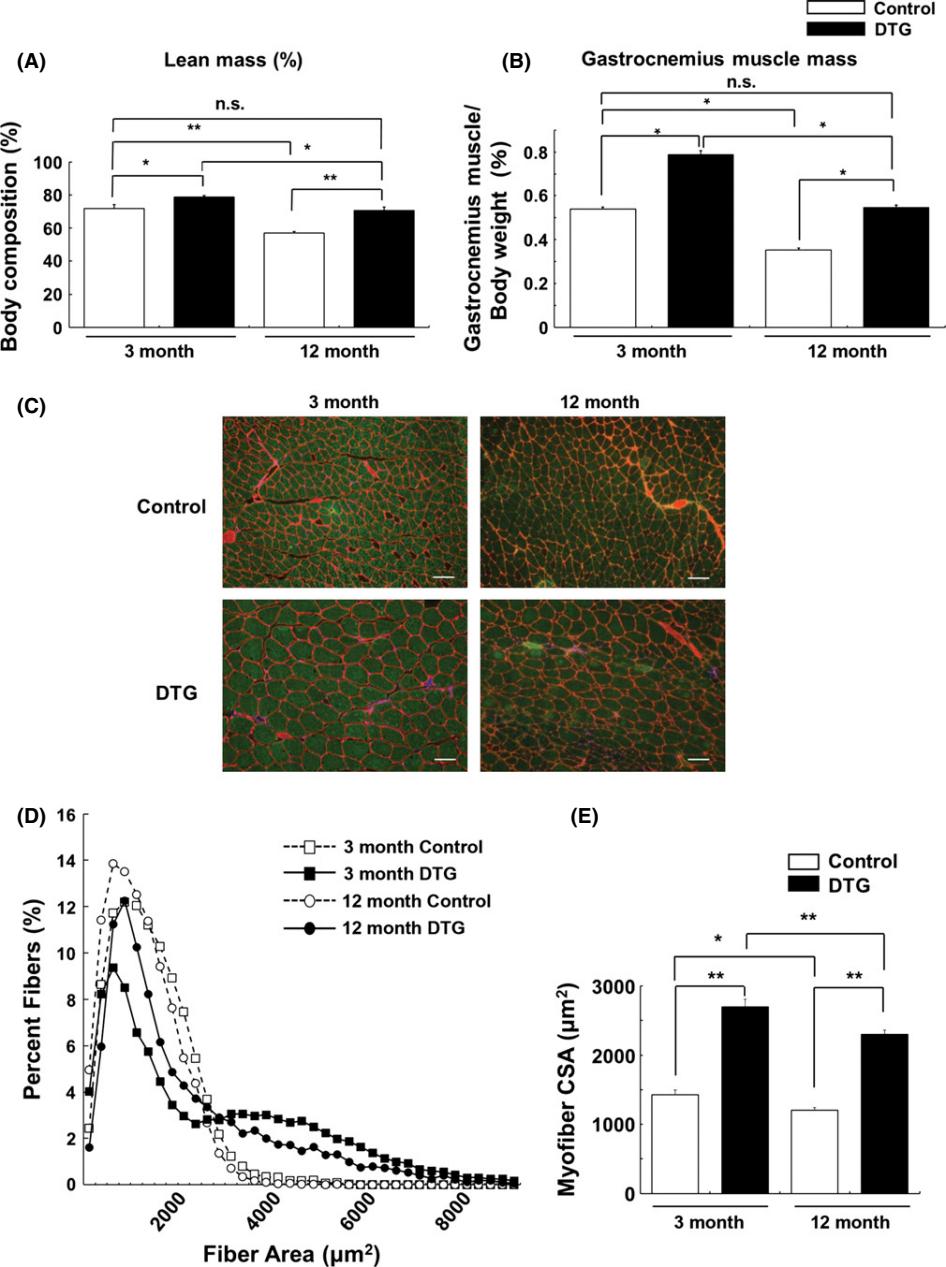
Akt1 activation increases lean mass and muscle weight in young and older mice. Body composition by quantitative magnetic resonance and gastrocnemius weight were measured in young and older control and double-transgenic (DTG) mice following 4 weeks of doxycycline administration. (A) Lean mass in experimental mice as a percentage of body weight (*n* = 7–10/group). (B) Gastrocnemius muscle of young and older mice expressed as a portion of total body weight. MCK-rtTA single transgenic mice were used as a control. Results are presented as mean ± SEM. *P < 0.05, **P < 0.01; n.s., not significant. (C) Induction of Akt1 in skeletal muscle increased type IIb myofiber size. Representative gastrocnemius muscle sections stained with MHC type IIb (green), laminin (red) and the nuclear stain DAPI (blue) from 3- and 12-month-old control and DTG mice after 4 weeks of Akt1 activation in skeletal muscle. Scale bars = 50 μm. (D) Distribution of mean cross-sectional area (CSA) of muscle fibers in gastrocnemius muscle in the different experimental groups. (E) Mean CSA of muscle fibers (*n* = 4/group). Results are presented as mean ± SEM. *P < 0.05, **P < 0.01.

In control mice, quantitative magnetic resonance (QMR) assessment revealed significantly less lean mass relative to body weight in 12-month-old mice compared with 3-month-old mice (Fig. 2A). Akt1 transgene activation in DTG mice increased lean mass in both age groups. In older mice, the induction of Akt1 transgene led to a level of lean mass that was indistinguishable from that of younger control mice. Analysis of gastrocnemius muscle weight in control mice showed that muscle weight of the 12-month-old mice was reduced compared with that of 3-month-old mice expressed either as muscle weight/body weight ratio (Fig. 2B) or in terms of absolute muscle weight (not shown). DOX treatment of DTG mice increased gastrocnemius muscle weight compared with DOX-treated control mice at both 3 and 12 months of age (1.5-fold and 1.4-fold, respectively). Although the overall extent of muscle growth in the older mice did not achieve the level seen in the younger DTG mice, the level of muscle mass in the transgenic 12-month-old mice closely matched that of the control 3-month-old mice.

Gastrocnemius muscle from the different experimental groups was processed for histology and stained with the plasma membrane marker laminin and type IIb myosin heavy chain isoform antibodies. Consistent with lower relative lean mass and muscle weight, quantitative analysis of muscle sections revealed that the average and overall size distributions of fibers of 12-month-old control mice were smaller than those in young mice (Fig. 2C–E). Activation of the Akt1 transgene in DTG young and old mice induced significant hypertrophy of type IIb myofibers (Fig. 2C). The extents of transgene-induced myofiber growth appeared to be heterogeneous, particularly in the older DTG mice, but the average increase in cross-sectional area was 1.9-fold in both young and older mice (Fig. 2E). Collectively, these data show that Akt1 transgene activation in skeletal muscle induced type IIb myofiber hypertrophy and increased lean mass in both younger and older animals.

### Activation of Akt signaling in muscle of young and middle-aged mice

**Figure 3:**
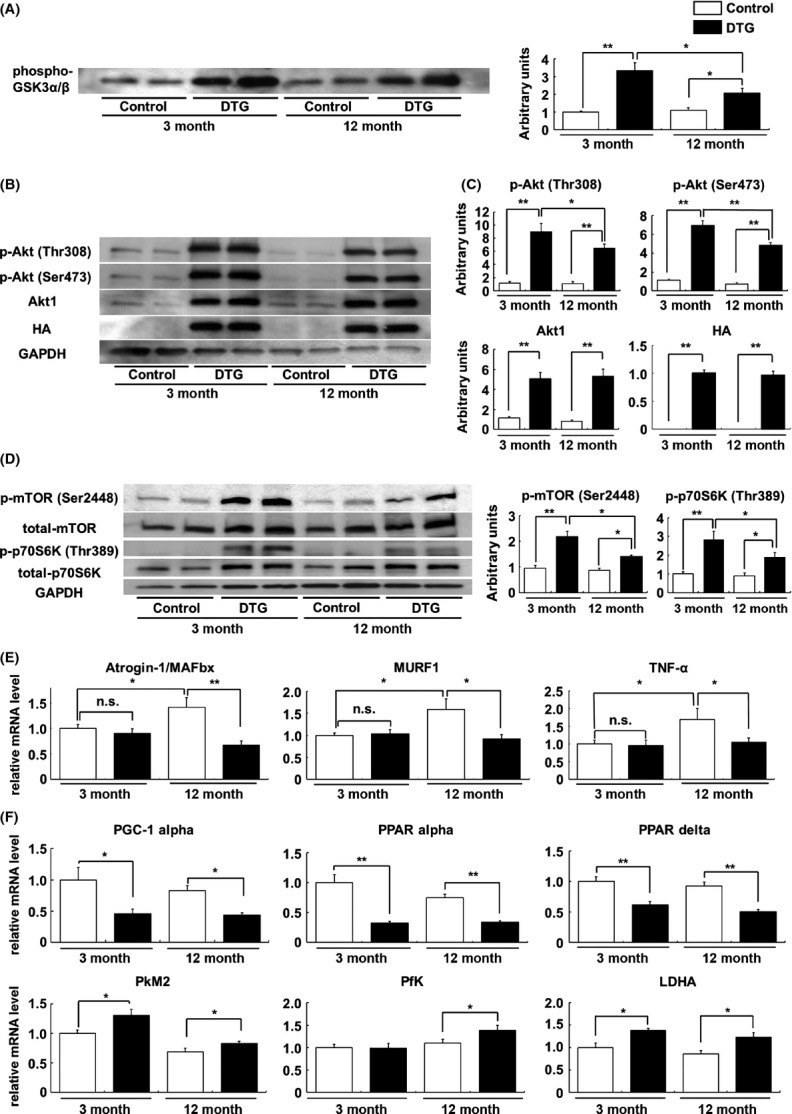
Transgene-mediated activation of Akt signaling in muscle. (A) Akt1 kinase activity in gastrocnemius muscle of 3- and 12-month-old control and double-transgenic mice after 4 weeks of doxycycline (DOX) treatment. MCK-rtTA single transgenic mice were used as controls. Top: representative GSK3α/β peptide phosphorylation. Bottom: histogram quantifying the data. *n* = 7/group. *P < 0.05, **P < 0.01. (B) Representative western blot analysis of Akt/PKB signaling. Phosphorylation of Akt at threonine 308 and serine 473, total Akt1 protein, Akt1 and transgene HA-tag was assessed in gastrocnemius muscle of the different experimental groups of mice. Representative blots are shown. HA, hemagglutinin. (C) Phosphorylation (p-Akt at Thr308 or Ser473) or protein levels (Akt1 or HA tag) were determined relative to GAPDH by Image J software analysis of autoradiography film (*n* = 7/group). Results are presented as mean ± SEM. *P < 0.05, **P < 0.01. (D) Regulation of downstream Akt effector molecules in muscle of young and older mice after 4 weeks of Akt transgene activation. Western blot analysis of downstream Akt targets mTOR and p70S6 kinase in gastrocnemius muscle. Representative blots of phosphorylation or total protein levels of mTOR and p70S6 kinase in control and double-transgenic (DTG) mice at 3 or 12 months of age. Phosphorylated levels of mTOR at Ser2448 or p70S6K at Thr389 were quantified relative to total mTOR or p70S6 kinase and then adjusted to GAPDH by Image J software analysis of autoradiography film (*n* = 7/group). (E) Relative mRNA expression levels of MAFbx, MURF, and TNF-α as measured relative to 36B4 by qRT-PCR in gastrocnemius muscle of control and DTG mice at 3 or 12 months of age (*n* = 7/group). Results are presented as mean ± SEM. *P < 0.05, **P < 0.01. (F) Myogenic activation of Akt signaling downregulates oxidative and upregulates glycolytic gene expression in muscle. Relative mRNA expression levels of PGC-1α, PPARα, PPARδ, pyruvate kinase isoenzyme M2 (PkM2), phosphofructokinase (Pfk), and LDHA as measured by qRT-PCR in gastrocnemius muscle of 3- or 12-month-old control and DTG mice after 4 weeks of DOX administration (*n* = 7/group). 36B4 was used as an internal control. Results are presented as mean ± SEM. *P < 0.05, **P < 0.01.

To assess the effect of aging and transgene induction on Akt1 activation and signaling in skeletal muscle, protein lysate was prepared from gastrocnemius muscle after 4 weeks of DOX treatment. An assay for Akt kinase activity demonstrated that although baseline Akt kinase activity did not differ between younger and older mice, consistent with the Akt Ser473 phosphorylation (Fig. 1), transgene induction led to approximately 3-fold and 2-fold increases in kinase activities in 3- and 12-month-old mice, respectively (Fig. 3A). Western blot analysis of gastrocnemius muscle revealed that administration of DOX markedly induced the levels of total Akt1 protein, Akt phosphorylation, and the expression of hemagglutinin (HA), the tag on the transgene in DTG mice (Fig. 3B). Quantitative protein expression showed that Akt1 and the HA tag were significantly increased in DTG mice, relative to glyceraldehyde 3-phosphate dehydrogenase (GAPDH), but there were no differences in the degree of induction between young and old animals (Fig. 3C). The phosphorylation of Akt at threonine 308 (Thr308) and serine 473 (Ser473), which are associated with its activation status, was markedly increased compared with control mice from both age groups. However, the degree of Akt phosphorylation in younger and older mice paralleled the degree of Akt kinase activity in the lysates of muscle (Fig. 3A), in that the younger mice appeared more responsive to transgene activation than the older mice (Fig. 3B,C).

Effector molecules downstream of Akt expression were also examined in the gastrocnemius muscle of young and middle-aged mice. Phosphorylation of the mammalian target of rapamycin (mTOR) at serine 2448 (Ser2448) and p70S6 kinase (p70S6K) at threonine 389 (Thr389) were significantly increased after 4 weeks of Akt1 transgene activation in animals of both age groups (Fig. 3D). As with the observed increases in Akt phosphorylation and activity, the increases in mTOR and p70S6K were greater in the 3-month-old DTG mice than in the 12-month-old mice.

### Akt-mediated changes in muscle gene expression in young and middle-aged mice

Catabolic pathways downstream of Akt1 were analyzed in gastrocnemius muscle of the different experimental groups of mice. Atrogin1/muscle atrophy F-box (MAFbx) and muscle ring finger 1 (MURF1) are ubiquitin ligases that mediate striated muscle atrophy (Sandri *et al*., 2004). Quantitative real-time PCR (qRT-PCR) revealed that Atrogin1/MAFbx and MURF expression increased in 12-month-old control mice compared with 3-month-old control mice (Fig. 3E). Akt1 transgene activation in 12-month-old DTG mice decreased the expression of these genes to levels observed in the muscle of 3-month-old mice. Interestingly, transcript levels of TNF-α, which upregulates Atrogin1/MAFbx and MURF1 expression in muscle (Li *et al*., 2005; Skurk *et al*., 2005; Frost *et al*., 2007), were increased in the gastrocnemius muscle of 12-month-old control mice. TNF-α expression in muscle was reduced to levels observed in young mice when the Akt1 transgene was activated in the older DTG mice. Collectively, these data indicate that Akt1 activation in skeletal muscle promotes anabolic and inhibits catabolic pathways, consistent with the development of a ‘resistance-trained’ phenotype.

A series of metabolic gene transcripts was also assessed in muscle of experimental mice. Gene expression was assessed by qRT-PCR in the gastrocnemius muscle of control and DTG mice at 3 and 12 months of age. Comparing young and older muscles of control mice did not reveal any statistically significant differences in the expression of genes associated with oxidative metabolism including peroxisome proliferator-activated receptor alpha (PPARα), delta (PPARδ) and gamma (PPARγ), and coactivator-1 alpha (PGC-1α) (Fig. 3F). In both young and older mice, activation of the Akt1 transgene led to statistically significant reductions in the levels of all of these genes. In contrast, Akt1 transgene induction led to increased expression of genes associated with glycolytic metabolism, including pyruvate kinase M2 (PkM2), phosphofructokinase (Pfk), and lactate dehydrogenase A (LDHA), in muscle of both young and middle-aged DTG mice (Fig. 3F). Collectively, these results indicate that Akt1 activation, in either young or older skeletal muscle, leads to an upregulation of glycolytic pathways and a downregulation of oxidative pathways in muscle, consistent with the selective growth of glycolytic type IIb fibers in this model (Izumiya *et al*., 2008).

### Akt-mediated muscle growth reverses age-associated increases in body weight and fat mass

**Figure 4:**
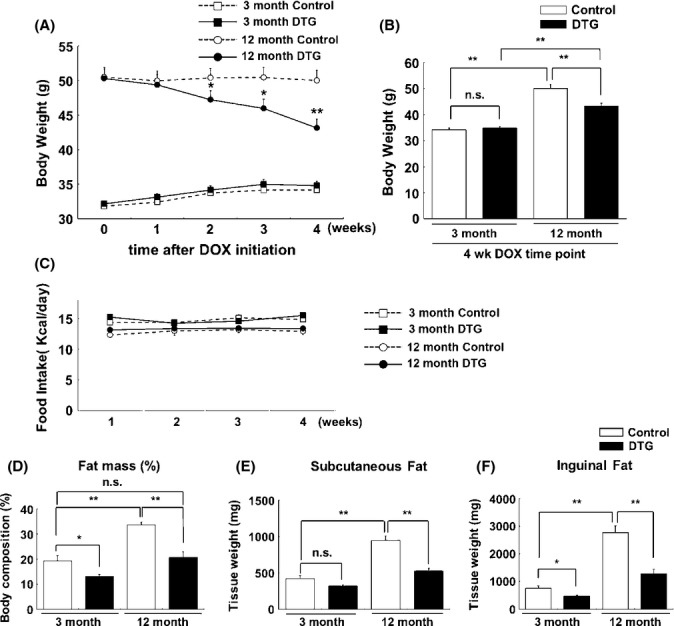
Age-associated accumulation of adipose tissue is reversed by restoration of glycolytic fast-twitch muscle. (A) Time course of body weight changes. Doxycycline (DOX) was administered to 3- or 12-month-old control and double-transgenic (DTG) mice to activate Akt1 in skeletal muscle. Body weight was measured weekly. MCK-rtTA single transgenic mice were used as controls. Results are presented as mean ± SEM. *P < 0.05, **P < 0.01. vs. 12-month-old control mice. (B) Body weight of control and DTG mice at the 4 week time point of DOX administration. (C) Food intake of young or aged control and DTG mice. DOX was given to activate Akt1 transgene in skeletal muscle for 4 weeks, and food intake was monitored weekly in 3- or 12-month-old control and DTG mice (*n* = 7–9/group). (D) Whole body composition of fat mass was measured by quantitative magnetic resonance in the different experimental groups. DOX was administered for 4 weeks (*n* = 13–15/group). Subcutaneous fat (E) and inguinal fat (F) weight after 4 weeks of DOX administration in young and older mice. Results are presented as mean ± SEM. *P < 0.05, **P < 0.01.

Total body weight of DTG and control mice was assessed prior to, and following, Akt1 transgene activation in muscle. Although mice were maintained on a standard low-fat chow diet, 12-month-old control mice were 49% larger in body weight compared with young mice (Fig. 4A). Transgene induction led to a time-dependent decrease in body weight in the older mice such that, by 4 weeks, the body weight of the older mice approached that observed in young mice (Fig. 4B). No significant differences were observed in the body weight of young mice following transgene activation. Age- and transgene-induction-induced changes in body weight could not be attributed to differences in food intake as young mice consumed slightly more food than old mice, and control and DTG mice within the same age group consumed equivalent quantities (Fig. 4C). Consistent with prior results (Izumiya *et al*., 2008), ambulatory activity of 3-month-old DTG mice was lower than that of 3-month-old control mice (Fig. S2). Ambulatory activity was also found to be reduced in 12-month-old control mice relative to 3-month-old control mice. Transgene activation in the older mice led to a trend toward reduced activity, but this was not statistically significant.

Quantitative magnetic resonance analysis revealed substantially greater overall fat mass of older control mice compared with young mice (Fig. 4D). Four weeks of Akt-mediated skeletal muscle growth in the older mice was associated with a marked reduction in fat mass, such that the levels of fat mass in older DTG mice were not significantly different from levels observed in young control mice. Akt transgene activation in young mice led to a small but statistically significant reduction in overall fat mass (Fig. 4D). This reduction in fat mass combined with an increase in lean muscle mass (Fig. 2A) may account for the lack of difference in body weight between 3-month-old control and DTG mice (Fig. 4A). In contrast, the loss of fat mass in the older mice exceeds the increase in lean muscle mass, leading to a net reduction in body weight. In the 12-month-old mice, analysis of adipose tissue weights revealed that activation of the Akt1 transgene in skeletal muscle led to 40% and 50% reductions in the masses of subcutaneous and inguinal fat, respectively (Fig. 4E,F). In contrast, in the younger mice, there was a modest loss of inguinal fat and a trend toward a reduction in subcutaneous fat that was not statistically significant.

A group of control and DTG mice were also analyzed at 18 months of age. Transgene activation for 4 weeks led to increases in overall lean mass, gastrocnemius muscle mass, and type IIb myofiber hypertrophy in the older mice (Fig. S1A–D). In this age group, Akt1 transgene activation in skeletal muscle led to reductions in overall fat mass, determined by QMR analysis, and reductions in subcutaneous and inguinal fat masses (Fig. S1E–G). However, the effect of transgene-induced muscle growth on fat mass reduction was less robust in 18-month-old mice compared with 12-month-old mice.

### Akt-mediated skeletal muscle growth improves metabolic parameters in middle-aged mice

**Figure 5:**
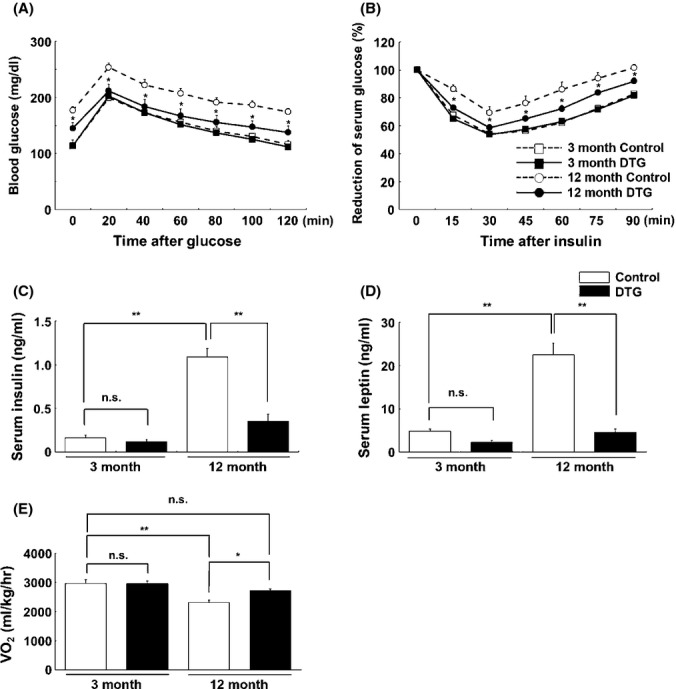
Myogenic Akt1 transgene activation corrects age-associated impairments in metabolism. (A) Glucose tolerance tests in 3- or 12-month-old control and double-transgenic (DTG) mice after 4 weeks doxycycline (DOX) treatment (*n* = 7–10/group). All mice were fasted overnight prior to measurements. Blood glucose was measured every 20 min for 2 h. *P < 0.05 vs. 12-month-old control mice. (B) Insulin tolerance tests in 3- or 12-month-old control and DTG mice after 4 weeks DOX treatment (*n* = 7–10/group). All mice were under 6-h fast condition. Blood glucose was measured every 15 min for 90 min. *P < 0.05 vs. 12-month-old control mice. Serum insulin (C) and serum leptin (D) levels in 3- or 12-month-old control and DTG mice after 4 weeks of DOX treatment (*n* = 8–10). Results are presented as mean ± SEM. *P < 0.05, **P < 0.01. (E) Akt1 activation increases whole body oxygen consumption. Young and older mice were monitored by Oxymax metabolic measuring system for 24 h under normal feeding conditions in control and DTG mice after 4 weeks administration of DOX (*n* = 6–7/group). Results are presented as mean ± SEM. *P < 0.05, **P < 0.01.

**Figure 6:**
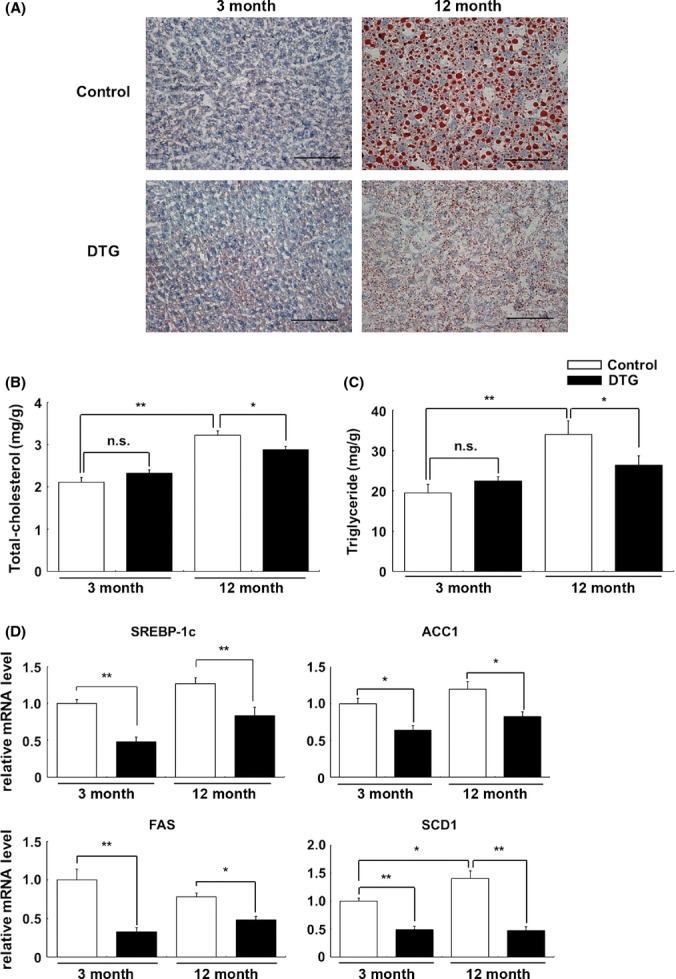
Myogenic activation of Akt1 signaling reduces hepatic steatosis. (A) Representative sections of liver stained with Oil red O in 3- or 12-month-old control and double-transgenic (DTG) mice after 4 weeks of doxycycline (DOX) administration. Scale bars = 100 μm. Quantitative analysis of total cholesterol (B) and triglyceride levels (C) in liver after 4 weeks DOX treatment in the different experimental groups (*n* = 8/group). Results are presented as mean ± SEM. *P < 0.05, **P < 0.01. (D) Myogenic activation of Akt1-signaling affects lipogenic gene expression. Relative mRNA expression levels of sterol regulatory element-binding protein (SREBP-1c), ACC1, FAS, and SCD1 were measured by qRT-PCR in liver of 3- or 12-month-old control and DTG mice after 4 weeks of DOX administration (*n* = 8/group). 36B4 was used as internal control. Results are presented as mean ± SEM. *P < 0.05, **P < 0.01.

Metabolic responses to glucose and insulin administration were assessed in 3- and 12-month-old control mice and corresponding DTG mice after 4 weeks of Akt1 transgene activation in skeletal muscle. Relative to younger mice, the older control mice displayed elevated glucose levels at baseline and both glucose intolerance and insulin resistance (Fig. 5A,B). These age-associated metabolic impairments were largely reversed by Akt1 activation in skeletal muscle. Transgene induction lowered resting glucose levels in older mice. Similarly, transgene activation improved glucose clearance following bolus administration of glucose or insulin in the 12-month-old mice. There was no effect of transgene activation in metabolically healthy young mice. Fasting serum insulin and leptin levels were also measured in the different experimental groups (Fig. 5C,D). Insulin and leptin levels were significantly higher in older than in younger control mice. Akt transgene activation in older mice led to marked reductions in serum insulin and leptin levels, returning their levels to that observed in the young mice (Fig. 5C,D). Whole body O_2_ consumption (VO_2_) was measured as an indicator of energy expenditure. VO_2_ did not differ between young control and DTG mice (Fig. 6A). However, 12-month-old control mice displayed significantly less energy expenditure than young control mice. After 4 weeks of Akt1 transgene activation in muscle, VO_2_ of middle-aged DTG mice was increased to an extent that nearly matched levels observed in young mice and likely contributed to the loss of fat mass. Collectively, these results indicate that even in the context of a standard low-fat chow diet, aging is associated with the development of insulin resistance and lowered energy expenditure and that the modest degree of Akt1-mediated transgene-induced growth of skeletal muscle is sufficient to return the metabolic state of mice to that observed in healthy young animals.

### Ectopic lipid accumulation with age is reversed by Akt-mediated muscle growth

Liver has important roles in regulating metabolic energy balance, and liver steatosis is a major contributor to insulin resistance. Thus, liver samples from the different experimental groups of mice were stained with Oil red O to assess ectopic lipid deposition. Strikingly, 12-month-old mice fed the standard low-fat chow diet displayed appreciable hepatic steatosis, whereas this was not observed in the young mice (Fig. 6A). In these older mice, 4 weeks of Akt1 transgene induction led to a dramatic reduction in the degree of visual lipid deposition. To quantify the degree of steatosis, total cholesterol (T-cho) and triglyceride (TG) contents of liver were examined (Fig. 6B,C). Consistent with the histological analysis, T-cho and TG levels were higher in the older mice, and these values returned to levels seen in the younger mice following Akt1 transgene activation in the muscle. Expressions of sterol regulatory element-binding protein 1c (SREBP-1c), acetyl-CoA carboxylase 1 (ACC1), fatty acid synthase (FAS), and stearoyl-CoA desaturase 1 (SCD1), which are involved in lipogenesis, were not significantly different between young and older mice, but were decreased by muscle-specific Akt1 transgene expression in both 3- and 12-month-old DTG mice (Fig. 6D). Thus, age-related alterations in hepatic gene expression and ectopic lipid accumulation associated with insulin resistance and T2DM are substantially reversed through skeletal-muscle-specific activation of Akt1 and the corresponding increase in muscle mass.

## Discussion

Here, we tested the extent to which changes in body composition contribute to metabolic dysfunction during the early stages of aging. Young (3 month old) and middle-aged (12 month old) mice, maintained in standard cages and on standard low-fat chow *ad libitum*, were analyzed for changes in body composition and systemic metabolism. The middle-aged mice displayed reductions in overall lean muscle mass, and an analysis of gastrocnemius muscle revealed reductions in tissue weight with a corresponding reduction in myofiber cross-sectional area. The older mice also displayed metabolic abnormalities including an impairment in the ability to clear glucose and the development of hepatic steatosis. These changes were associated with elevated levels of circulating leptin and insulin. The older mice in this study also displayed a state of anabolic resistance. Although unstimulated levels of signaling intermediates in muscle, including Akt, mTOR, and 70S6K phosphorylation, were not different between the 3- and 12-month-old mice, the intracellular signaling response to IGF-1 administration was markedly different between these age groups. These observations highlight the concept that aging promotes a state of both anabolic and metabolic resistance.

Because Akt is a central mediator of both anabolic and metabolic pathways, we tested whether the short-term activation of this signaling step could overcome the age-dependent block in this pathway, thereby reversing the decline in lean muscle mass and promoting metabolic function. In this model, the genetic activation of myristoylated Akt is mediated by the 1256[3Emut] MCK promoter fragment that is expressed in a subset of skeletal muscle (e.g., gastrocnemius and quadriceps), but not in soleus or extensor digitorum longus (Izumiya *et al*., 2008). Thus, while some muscle groups display considerable hypertrophy, the overall increase in whole body skeletal muscle mass is relatively modest in this model. As such, the reduction in relative lean muscle mass that occurs by 12 months of age is closely matched with the transgene-induced growth of skeletal muscle, and there is no statistically significant difference in the levels of lean muscle mass relative to body weight between 3-month-old control mice and the 12-month-old transgenic mice. In effect, this system allowed us to assess the consequences of restoring the lean muscle mass that is lost by the 12-month time point. Furthermore, this inducible transgenic model leads to the selective growth of glycolytic, fast-twitch fibers that are preferentially lost during the aging process (Larsson, 1983; Lexell *et al*., 1983).

Aging in this model was associated with increased expression of factors associated with muscle atrophy and inflammation including atrogin-1/MAFbx, MURF1, and TNF-α. Acute Akt transgene expression led to reductions in their expression of these factors, indicative of a reversal of the molecular phenotype of the aging muscle. Akt-mediated skeletal muscle growth also restored a functional metabolic phenotype in the 12-month-old mice. Metabolic improvement was indicated by increased glucose tolerance and insulin sensitivity and a marked resolution of hepatic steatosis. The metabolic normalization was also indicated by reductions in circulating levels of insulin and leptin, and by changes in the metabolic transcript profile in liver. The increase in lean muscle mass was coupled to a reversal in adipose tissue mass that was acquired over 12 months in the mice fed a low-fat standard chow diet. Similarly, we observed that an Akt-mediated increase in lean muscle mass led to a reduction in fat mass in 18-month-old mice. However, these changes were not as robust as in the 12-month-old mice, perhaps because the transgene-induced increase in muscle could not completely compensate for the loss of lean mass that occurs at the more advanced age. Collectively, these data show that the Akt-mediated restoration of glycolytic skeletal muscle in middle-aged mice is sufficient to reverse the metabolic profile to that of young animals.

We speculate that age-associated anabolic resistance underlies the observed changes in skeletal muscle mass and insulin sensitivity in older mice. Whereas the PI3K-/Akt-/mTOR-signaling pathway is responsive to anabolic signals in young mice, this is not the case in older mice, and the increased activation of Akt through transgene expression brings these activities to the levels that are observed in young mice. Importantly, these data do not support the notion that the loss in lean muscle mass and metabolic dysfunction are independent epiphenomena of the aging process. Instead, the results suggest that the loss of muscle mass is causally related to age-associated metabolic dysfunction. Based upon this rodent study, it is tempting to speculate that the surge in newly diagnosed cases of T2DM seen in midlife (Centers for Disease Control and Prevention, 2011) may be in large part attributable to the loss in glycolytic muscle mass that occurs within this time frame.

Activation of the Akt1 transgene led to the selective growth of glycolytic (type IIb) fibers in mice [(Izumiya *et al*., 2008) and Fig. 3]. In contrast, most studies have focused on the metabolic aspects of oxidative muscle (types I, IIa, and IIx), where the PGC-1 transcriptional regulator of mitochondrial biogenesis appears to predominate. In this regard, there are conflicting reports about the systemic metabolic regulatory properties of the oxidative muscle phenotype that is induced by PGC-1. Transgenic overexpression of PGC1α will increase mitochondrial content of muscle and improve running capacity, but it has been reported that these mice are not protected from increases in body weight and fat weight, and that they are more prone to insulin resistance when fed a high calorie diet (Choi *et al*., 2008). On the other hand, it has been shown that a modest level of PGC-1α overexpression in skeletal muscle can increase insulin sensitivity (Benton *et al*., 2008) and that it protects against muscle loss and metabolic dysfunction upon aging (Wenz *et al*., 2009). However, it is striking that the combined ablation of PGC-1α and PGC-1β in muscle has no effect on insulin sensitivity and glucose tolerance (Zechner *et al*., 2010), and that the ablation of PGC-1α in muscle has no impact on the metabolic benefits of caloric restriction (Finley *et al*., 2012), despite findings of impaired mitochondrial biogenesis in both of these models. These observations and others have led some to question the role of skeletal muscle mitochondria in insulin resistance (Holloszy, 2009). Because the muscle of patients with T2DM contain ~30% fewer mitochondria than those with normal insulin sensitivity (He *et al*., 2001; Kelley *et al*., 2002), the notion that ‘mitochondrial deficiency’ is causally linked to metabolic dysfunction has become popular. In contrast, it has been suggested that skeletal muscle mitochondria are in vast excess of what is required to oxidize fatty acids to supply energy, such that a state of mitochondrial deficiency is unlikely to develop (Holloszy, 2009). Furthermore, it has been reported that high fat diets increase muscle mitochondria content concomitant with metabolic dysfunction (Hancock *et al*., 2008).

With regard to the considerations discussed above, transgenic overexpression of Akt leads to a decrease in mitochondrial markers in skeletal muscle, both in aging mice and young mice on high caloric diet (Izumiya *et al*., 2008), yet both of these models display marked metabolic improvements following transgene activation. Thus, we attribute these improvements in metabolism to the increase in glycolytic muscle mass. Overall energy expenditure is directly related to the level of lean body mass (Reitman, 2002), as a result of the energy demand associated with maintaining myofibrillar structures. Thus, we speculate that the loss of glycolytic muscle, which occurs upon aging, leads to an ‘energy expenditure deficiency’ that contributes to metabolic dysfunction. Consistent with this notion, an increase in lean muscle mass in 12-month-old mice leads to an increase in energy expenditure, such that aging mice with Akt transgene activation display VO_2_ levels that are comparable with those seen in young mice. Of particular interest, this increase in energy expenditure occurs despite reductions in markers of fatty acid oxidation and mitochondrial biogenesis in the muscle. Based upon these findings, we propose that the expansion of muscle mass through hypertrophy of glycolytic, fast-twitch fibers puts an increasing energy burden on remote tissues, contributing to the resolution of metabolic dysfunction in the aging animal. In partial support of this hypothesis, we observed that Akt-mediated skeletal muscle growth led to the inhibition of lipogenic genes and resolved the hepatic steatosis that developed by 12 months of age. An alternative mechanism is that the increase in lean muscle mass in the older mice will lead to greater carbohydrate utilization by the glycolytic muscle as opposed to its storage as fat in adipose tissue.

In summary, we show that the early stages of murine aging are associated with the atrophy of type IIb myofibers, the development of anabolic resistance in muscle, and the development of systemic metabolic dysfunction. The replenishment of type IIb fiber mass, through elevated Akt1 signaling, is sufficient to reverse the decline in metabolic function, suggesting that the age-associated loss in glycolytic, fast-twitch muscle mass may be causal for the metabolic abnormalities that appear in mid-life. Work with this model system supports the concept that exercise regimens, such as resistance training, or agents that promote glycolytic myofiber hypertrophy may be particularly efficacious in promoting metabolic health in the aging population.

## Experimental procedures

### Skeletal muscle-specific Akt1 transgenic mice

Thousand two hundred and fifty six [3Emut] MCK-rtTA transgenic mice and TRE-myrAkt1 transgenic mice were crossed to generate DTG mice (Shiojima *et al*., 2005). DTG mice were given DOX (0.5 mg mL^−1^) in drinking water, which activates *Akt1* transgene in DTG mice. In all experiments, 3- or 12-month-old mice were treated with DOX for 4 weeks. 1256 [3Emut] MCK-rtTA single transgenic and same age littermates were used as controls and DOX was administered to all experimental groups of mice. Study protocols were approved by the Boston University Institutional Animal Care and Use Committee. Mice were fed a standard chow diet (Harlan Teklad global 18% protein rodent diet, #2018) and monitored food consumption and body weight daily in individual cages.

### Intramuscular injection of IGF-1

Mice, 3- or 12 month old, were injected with either vehicle (sterile water) or human IGF-1 directly into the gastrocnemius of the right leg using a 29-gauge insulin syringe. Mice were sacrificed 1 h postdose, and the gastrocnemius was removed and snap-frozen in liquid nitrogen. Human IGF-1 was purchased from Peprotech Inc (Rocky Hill, NJ, USA).

### Physiological measurements

O_2_ consumption and CO_2_ production were measured using an eight-chamber Oxymax/Comprehensive Lab Animal Monitoring System (Columbus Instruments, Columbus, OH, USA) with one mouse per chamber. This system simultaneously measures physical activity. All mice were acclimated to monitoring chambers for 2 h before the experiment, and food and water were given *ad libitum*. During the first 24 h, mice were analyzed under fed conditions and without food for the following 24 h. All cages were kept at 24 °C and under a 12 h light–dark cycle. Food intake was measured 2 days per week for 4 weeks.

### Body composition and metabolic measurements

EchoMRI-900 quantitative nuclear magnetic resonance (NMR) system (Echo Medical Systems, Houston, TX, USA) was used for determination of lean mass and fat mass after 4 weeks DOX treatment. This noninvasive measure was performed on conscious mice. Glucose tolerance tests were performed on overnight-fasted mice injected i.p. with D-glucose (1 g kg^−1^ body weight). Tail blood was collected immediately before and at intervals after the injection. Insulin tolerance tests were performed on 6 h-fasted mice injected i.p. with human insulin (0.75 U kg^−1^ body weight, Humulin R, Eli Lilly). Tail blood was collected immediately before and at intervals after injection. Blood glucose was measured by an Accu-Chek glucose monitor (Roche Diagnostics Corp., Basel, Switzerland). Serum insulin and leptin levels were determined by ELISA (Crystal Chemical Inc., Downers Grove, IL, USA).

### Histology

2-Methylbutane and liquid nitrogen were used for skeletal muscle fixation. Liver tissues were embedded in OCT compound (Sakura Finetech USA Inc., Torrance, CA, USA) and snap-frozen in liquid nitrogen. Liver tissue sections were stained with haematoxylin and eosin for overall morphology and Oil red O for lipid deposition by standard methods. Myofibers were stained with antilaminin antibody (Neomarkers, Fremont, CA, USA), and type IIb fibers were detected with MHC type IIb monoclonal antibody, which is the supernatant of the BF-F3 hybridoma cell line. The cross-sectional areas of muscle fibers were calculated using MetaMorph software (Sunnyvale, CA, USA).

### Western blot and Akt kinase assay

Western blot analysis was performed as described previously (Shiojima *et al*., 2002). Antibodies used were phospho-Akt (Ser473), phospho-mTOR (Ser2448), mTOR, phospho-p70 S6K (Thr389), and GAPDH from Cell Signaling Technology (Danvers, MA, USA); Akt1, HA tag, and p70 S6K from Santa Cruz Biotechnology (Dallas, TX, USA). Akt kinase activity was measured with an Akt kinase assay kit from Cell Signaling Technology. Briefly, phospho-Akt (Ser473) monoclonal antibody was used to immunoprecipitate Akt from protein lysate of gastrocnemius muscle. *In vitro* kinase assay was performed using GSK-3 fusion protein as substrate. The phosphorylation of GSK-3 was detected using phospho-GSK-3(α/β)(Ser21/9) antibody. ECL Plus Western Blotting Detection Reagents (Amersham) were used for detection. Phosphorylation or protein levels were determined by Image J software (NIH, Bethesda, MD, USA). GAPDH was used as internal control.

### Quantitative real-time PCR

Total RNA from gastrocnemius muscle and liver was isolated with RNeasy kit (Qiagen, Venlo, The Netherlands), and cDNA was synthesized by Superscript III First strand Synthesis System (Invitrogen, Carlsbad, CA, USA). Real-time PCR was performed with StepOne™ Real-Time PCR System using SYBR Green PCR Master Mix (Applied Biosystems, Carlsbad, CA, USA). Transcript levels were determined as the number of transcripts relative to those of 36B4 and additionally normalized to the mean value of gastrocnemius muscle or liver from 3-month-old control mice. Primer sequences are listed in Table S1.

### Liver lipid analysis

Briefly, snap-frozen liver (100 mg) was homogenized in 2 mL of chloroform/methanol (2:1 v/v) solution. 2 mL of 50 mm NaCl was added and centrifuged for 10 min at 1500 RCF. The organic phase was dried using N_2_ gas and resolubilized in 2-propanol containing 10% Triton X-100. T-cho and TG levels were determined by enzymatic kits (Wako Diagnostics, Richmond, VA, USA).

### Statistical analysis

All data are expressed as mean ± SEM. Statistical comparisons of data were made by anova with Tukey–Kramer method.

## Supporting Information

**Fig. S1** Akt1-mediated increases in lean muscle mass in 18-month-old mice reduces age-associated accumulation of adipose tissue. **Fig. S2** Spontaneous ambulatory activity levels were measured in the different experimental groups by the infrared beam interruption method.

**Table S1** Primers used for qRT-PCR protocols.
