# Supplementary material for: Glycolytic fast-twitch muscle fiber restoration counters adverse age-related changes in body composition and metabolism
Source: Aging Cell. 2013 Sep 17;13(1):80–91. doi: 10.1111/acel.12153 (PMC3947044; doi:10.1111/acel.12153)
Supplement: Supplementary file 1 — Fig. S1 Akt1-mediated increases in lean muscle mass in 18-month-old mice reduces age-associated accumulation of adipose tissue. Fig. S2 Spontaneous ambulatory activity levels were measured in the different experimental groups by the infrared beam interruption method. [file acel0013-0080-sd1.pdf]

Supporting Information

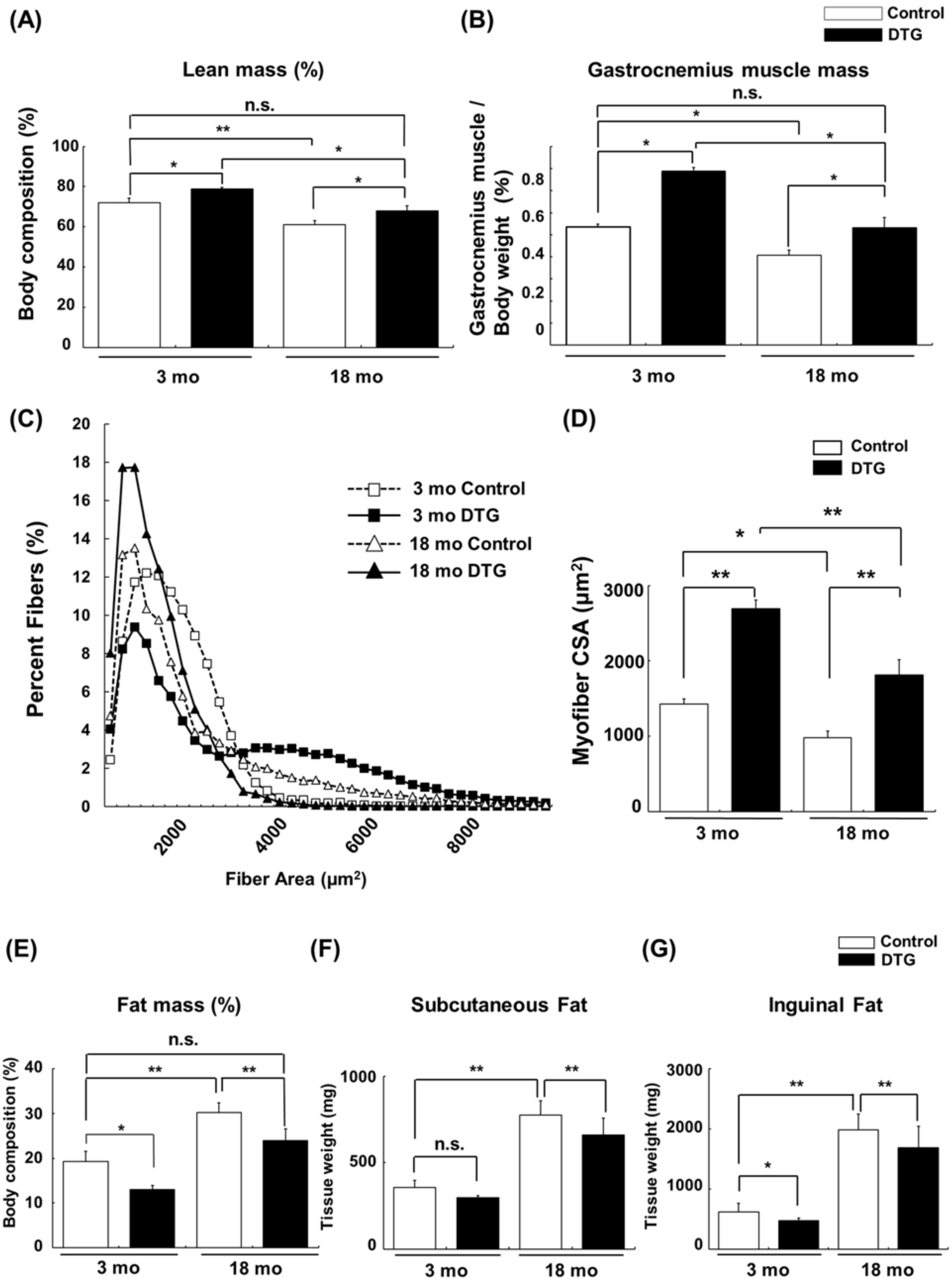
(A)
Lean mass (%)
Body composition (%)
100
80
60
40
20
0
n.s.
**
*
*
*
3 mo
18 mo
(B)
Control
DTG
Gastrocnemius muscle mass
Gastrocnemius muscle / Body weight (%)
0.8
0.6
0.4
0.2
0
n.s.
*
*
*
*
3 mo
18 mo
(C)
Percent Fibers (%)
20
18
16
14
12
10
8
6
4
2
0
3 mo Control
3 mo DTG
18 mo Control
18 mo DTG
2000
4000
6000
8000
Fiber Area (μm2)
(D)
Control
DTG
Myofiber CSA (μm2)
3000
2000
1000
0
*
**
**
**
3 mo
18 mo
(E)
Fat mass (%)
Body composition (%)
40
30
20
10
0
n.s.
**
**
*
3 mo
18 mo
(F)
Subcutaneous Fat
Tissue weight (mg)
1000
500
0
**
**
n.s.
3 mo
18 mo
(G)
Control
DTG
Inguinal Fat
Tissue weight (mg)
3000
2000
1000
0
**
**
*
3 mo
18 mo

Supplemental Figure 1. Akt1-mediated increases in lean muscle mass in 18-month old mice reduces age-associated accumulation of adipose tissue. (A) Whole body composition of lean mass was measured by quantitative magnetic resonance following 4 weeks of doxycycline administration. Lean mass in experimental mice is expressed as a percentage of total body weight (n=8-10 in each group). MCK-rtTA single transgenic mice were used as a control. (B) Gastrocnemius muscle weight is expressed as a portion of total body weight in the different experimental groups. (C) Distribution of mean cross-sectional area (CSA) of muscle fibers in gastrocnemius muscle in the different experimental groups. (D) Mean CSA of muscle fibers determined from the data in C (n=4 in each group). (E) Whole body composition of fat mass was measured by quantitative magnetic resonance in the different experimental groups. (n=8-10 in each group). Subcutaneous fat (F) and inguinal fat (G) weight after 4 weeks of DOX administration in 3-month and 18-month old mice. Results are presented as mean ± SEM. *P<0.05, **P<0.01. The 3-month-old data are the same as in the main text of the manuscript, and they are shown here for comparison.

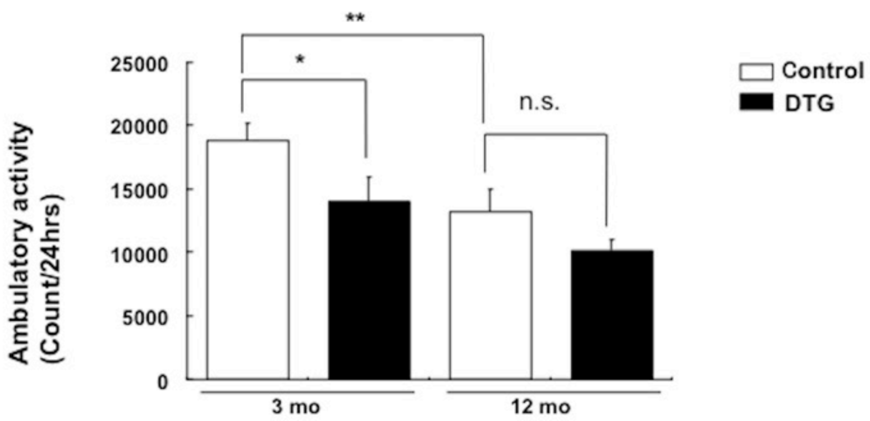

Supplemental Figure 2. Spontaneous ambulatory activity levels were measured in the different experimental groups by the infrared beam interruption method. Photo cells aligned on the x, y and z axes of metabolic chambers were used to measure activity. Results are presented as mean ± SEM. *P<0.05, **P<0.01, n.s. = not significant.
