# Supplementary material for: Glycolytic fast-twitch muscle fiber restoration counters adverse age-related changes in body composition and metabolism
Source: Aging Cell. 2013 Sep 17;13(1):80–91. doi: 10.1111/acel.12153 (PMC3947044; doi:10.1111/acel.12153)
Supplement: Supplementary file 2 — Table S1 Primers used for qRT-PCR protocols. [file acel0013-0080-sd2.pdf]

Supplement Table 1. Primers used for qRT-PCR protocols.

Gene	Primer Sequence	
Atrogin-1/MAFbx	Forward	5’-ATGCACACTGGTGCAGAGAG-3’
	Reverse	5’-TGTAAGCACACAGGCAGGTC-3’
MURF1	Forward	5’-CAGGGGACGAAGACAAAGAG-3’
	Reverse	5’-GGTTCTCCATAGCGTTTCCA-3’
TNF-α	Forward	5’-CATCTTCTCAAAATTCGAGTGACAA-3’
	Reverse	5’-TGGGAGTAGACAAGGTACAACCC-3’
PGC-1α	Forward	5'-AAAACAGGAACAGCAGCAGAGAGAC-3'
	Reverse	5'-GGGGTCAGAGGAAGAGATAAAGTTG-3'
PPARα	Forward	5'-CAATCCCCTCCTGCAACTTC-3'
	Reverse	5'-ATTTCCCTGTTTGTGGCTGCTA-3'
PPARδ	Forward	5'-TCACCGGCAAGTCCAGCCA-3'
	Reverse	5'-ACACCAGGCCCTTCTCTGCCT-3'
PkM2	Forward	5’-ATTGGGCCTGCTTCCCGATC-3’
	Reverse	5’-CAAACCTGCGGACGCCTTCAT-3’
Pfk	Forward	5'-GGCCAATCCTCAAAATCCTA-3'
	Reverse	5'-CCAGACCGTTTCCTTGAAAT-3'
LDHA	Forward	5'-TGTGTGGAGTGGTGTGAATG-3'
	Reverse	5'-ACCTGCTTGTGAACCTCCTT-3'
SREBP-1c	Forward	5’-CAAAACCAGCCTCCCAAGA-3’
	Reverse	5’-TCCCCGTCCACAAAGAAAC-3’
ACC1	Forward	5’-GTTGCACAAAAGGATTTCA-3’
	Reverse	5’-CGCATTACCATGCTCCGC-3’
FAS	Forward	5’-GTGTGGTGGGTTTGGTGAA-3’
	Reverse	5’-GATGTGTTGCTGAGGTTGGA-3’
SCD1	Forward	5'-CAAGCTGGAGTACGTCTGGA-3'
	Reverse	5'-CAGAGCGCTGGTCATGTAGT-3'
36B4	Forward	5'-GCTCCAAGCAGATGCAGCA-3'
	Reverse	5'-CCGGATGTGAGGCAGCAG-3'
